# Revealing the Sole Impact of Acceptor's Molecular Conformation to Energy Loss and Device Performance of Organic Solar Cells through Positional Isomers

**DOI:** 10.1002/advs.202103428

**Published:** 2022-03-24

**Authors:** Guilong Cai, Zeng Chen, Mengyang Li, Yuhao Li, Peiyao Xue, Qingbin Cao, Weijie Chi, Heng Liu, Xinxin Xia, Qiaoshi An, Zheng Tang, Haiming Zhu, Xiaowei Zhan, Xinhui Lu

**Affiliations:** ^1^ Department of Physics The Chinese University of Hong Kong New Territories Hong Kong 999077 China; ^2^ State Key Laboratory of Modern Optical Instrumentation Center for Chemistry of High‐Performance & Novel Materials Department of Chemistry Zhejiang University Hangzhou Zhejiang 310030 China; ^3^ Center for Advanced Low‐dimension Materials State Key Laboratory for Modi cation of Chemical Fibers and Polymer Materials College of Materials Science and Engineering Donghua University Shanghai 201620 China; ^4^ School of Materials Science and Engineering Peking University Beijing 100871 China; ^5^ School of Chemistry and Chemical Engineering Beijing Institute of Technology Beijing 100081 China; ^6^ Fluorescence Research Group Singapore University of Technology and Design Singapore 487372 Singapore

**Keywords:** bridge effect, fused‐ring electron acceptors, nonfullerene acceptors, organic solar cells, positional isomer

## Abstract

Two new fused‐ring electron acceptor (FREA) isomers with nonlinear and linear molecular conformation, *m*‐BAIDIC and *p*‐BAIDIC, are designed and synthesized. Despite the similar light absorption range and energy levels, the two isomers exhibit distinct electron reorganization energies and molecular packing motifs, which are directly related to the molecular conformation. Compared with the nonlinear acceptor, the linear *p*‐BAIDIC shows more ordered molecular packing and higher crystallinity. Furthermore, *p*‐BAIDIC‐based devices exhibit reduced nonradiative energy loss and improved charge transport mobilities. It is beneficial to enhance the open‐circuit voltage (*V*_OC_) and short‐current current density (*J*_SC_) of the devices. Therefore, the linear FREA, *p*‐BAIDIC yields a relatively higher efficiency of 7.71% in the binary device with PM6, in comparison with the nonlinear *m*‐BAIDIC. When *p*‐BAIDIC is incorporated into the binary PM6/BO‐4Cl system to form a ternary system, synergistic enhancements in *V*_OC_, *J*_SC_, fill factor (FF), and ultimately a high efficiency of 17.6% are achieved.

## Graphical abstract

The sole impact of molecular conformation to device performance is studied by the design of two structural isomers, which suggests that the molecular linearity directly affects the molecular packing and electron reorganization energy, and thus being critical to energy loss and device performance.

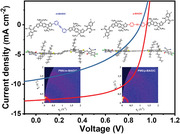

## 1 Introduction

Organic solar cells (OSCs), which offer unique advantages, such as low cost, light weight, semitransparency, flexibility, and solution fabrication, have been studied extensively nowadays.^[^ ^1^, ^2^, ^3^, ^4^ ^]^ They employ bulk heterojunction in the active layer to increase the exciton dissociation and charge collection efficiencies due to abundant donor/acceptor (D/A) interfaces and intercalated charge transport network.^[^ ^5^, ^6^ ^]^ Consequently, the conformation of the molecule has a significant impact on the intermolecular interaction and thus the bulk morphology, which will be reflected in device performance ultimately.^[^ ^7^, ^8^ ^]^ Therefore, the development of new materials (e.g., donor, acceptor, and electron/hole transport materials) is key to enhancing the device performance, in terms of improving the photoelectric properties as well as bulk morphology.^[^ ^9^, ^10^, ^11^, ^12^, ^13^, ^14^, ^15^, ^16^ ^]^ In recent years, breakthroughs in new acceptors are leading the advancement of OSC efficiencies, which were launched with the invention of 3,9‐bis(2‐methylene‐(3‐(1,1‐dicyanomethylene)‐indanone)‐5,5,11,11‐tetrakis(4‐hexylphenyl)‐dithieno[2,3‐d:2′,3′‐d′]‐s‐indaceno[1,2‐b:5,6‐b′]‐dithiophene (ITIC)—the first high‐performance fused‐ring electron acceptors (FREAs) synthesized by Zhan and co‐workers.^[^ ^9^ ^]^ Impressively, the synthesis of Y6^[^ ^17^ ^]^ and the development of Y‐series acceptor materials further pushed the power conversion efficiencies (PCE) of OSCs over 18%.^[^ ^13^, ^18^, ^19^, ^20^, ^21^, ^22^, ^23^, ^24^, ^25^, ^26^ ^]^

To date, lots of FREAs have been designed and synthesized to increase the open‐circuit voltage (*V*_OC_) and short‐current current density (*J*_SC_) by modulating energy levels and light absorption range.^[^ ^27^, ^28^, ^29^, ^30^, ^31^, ^32^, ^33^, ^34^, ^35^, ^36^, ^37^, ^38^, ^39^, ^40^, ^41^, ^42^, ^43^, ^44^, ^45^, ^46^, ^47^ ^]^ However, the efficiency of OSCs still lags behind crystalline silicon and perovskite solar cells, which is mainly attributed to the large energy loss in active layer. It is suggested that the energy loss is closely related to the molecular packing motif.^[^ ^26^, ^48^ ^]^ Thus, studies have shown that the energy loss can be effectively reduced by the modification of the acceptor central core,^[^ ^49^ ^]^ end groups,^[^ ^40^, ^50^ ^]^ and side chains^[^ ^25^, ^51^ ^]^ as well as through ternary strategies.^[^ ^22^, ^52^ ^]^ It is worth noting that the influence of the steric configuration of the acceptor molecule on device performance has been demonstrated. However, the sole impact of the molecular conformation on the device performance is not fully understood yet, because it is challenging to rule out the influence of light absorption and energy level changes while modifying the molecular design.^[^ ^53^, ^54^, ^55^, ^56^ ^]^ Fortunately, our previous work demonstrated that dimer strategy has great potential in the design of molecular conformation compared to the monomer acceptors due to the extensive linking units and simple synthesis process.^[^ ^37^, ^57^ ^]^ On the one hand, the dimer with acetylene unit link displays a high electron mobility and preferential face‐on molecular packing. On the other hand, the appropriate introduction of benzenes in the linking unit not only can change the molecular conformation but also can potentially up‐shift the lowest unoccupied molecular orbital (LUMO) level, thus improving the *V*_OC_.^[^ ^34^, ^58^ ^]^

**Figure 1:**
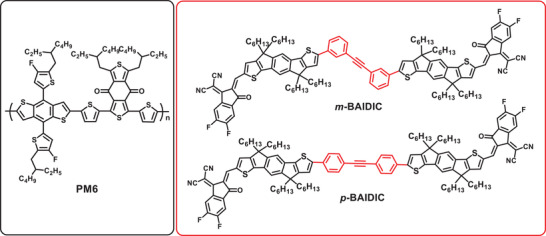
Chemical structures of PM6, *m*‐BAIDIC, and *p*‐BAIDIC.

Hence, in this work, two new FREA isomers with different molecular conformations, named *m*‐BAIDIC and *p*‐BAIDIC, were designed and synthesized based on indaceno[3,2‐*b*]dithiophene dimers bridged by *m*/*p*‐diphenylacetylene, respectively (**Figure** **1**). Both light absorption and energy levels of the two isomers are highly similar with each other. As a result, the sole impact of the molecular conformation on the device performance can be investigated. First, the linear *p*‐BAIDIC exhibits lower electron reorganization energy, higher electron mobility (*μ*_e_), and more ordered molecular packing than the nonlinear *m*‐BAIDIC. When a mid‐bandgap polymer PM6 is adopted as donor, the optimized *p*‐BAIDIC‐based device yields a much higher efficiency of 7.71%. In contrast, the *m*‐BAIDIC‐based counterpart exhibits much worse performance in terms of all the device characteristics. Particularly, the *p*‐BAIDIC‐based device shows a higher *V*_OC_ of 0.053 V than that the *m*‐BAIDIC‐based device, reflecting the significant influence of molecular conformation on the energy loss. Furthermore, ternary blend strategy is widely recognized as complementary to the binary system in terms of energy level, morphology, and light absorption, thus effectively improving device performance.^[^ ^59^ ^]^ Therefore, we introduced the *p*‐BAIDIC into PM6/BO‐4Cl binary OSCs to fabricate the ternary system. The *p*‐BAIDIC‐based ternary OSC displays a champion PCE of 17.6%, and the ternary system effectively reduces the energy loss and synergistically enhances the *V*_OC_, *J*_SC_, and fill factor (FF) compared to the PM6/BO‐4Cl binary control.

## 2 Results and Discussion

### 2.1 Synthesis and Optoelectronic Properties

Two positional isomers (*m*‐BAIDIC and *p*‐BAIDIC) with excellent solubility in common organic solvents (chloroform and chlorobenzene) were carefully synthesized, and the synthetic routes are shown in Scheme S1 in the Supporting Information.

**Figure 2:**
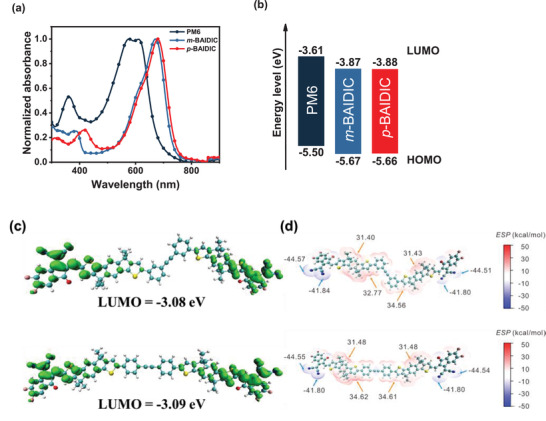
a) UV‐vis absorption spectra and b) energy levels of PM6, *m*‐BAIDIC, and *p*‐BAIDIC in thin films; c) the optimal geometries, LUMO distribution; and d) ESP of *m*‐BAIDIC and *p*‐BAIDIC.

Both *m*‐BAIDIC and *p*‐BAIDIC show strong light absorbance in 550–750 nm region with the corresponding molar attenuation coefficients of 1.1 × 10^5^ and 1.4 × 10^5^ M^−1^ cm^−1^ in dilute solution (10^−6^ m) (Figure S1, Supporting Information), indicating that although *p*‐BAIDIC has slightly stronger absorption in the visible region, the two dimers have similar absorption profiles and optical bandgaps (*E*_g_^opt^ = 1.66–1.68 eV) in thin films (**Figure** **2**a and **Table** **1**). As expected, the two acceptors also have similar highest occupied molecular orbital (HOMO) and LUMO energy levels estimated by cyclic voltammetry (CV; Figure S2, Supporting Information and Table 1), forming appropriate energy level alignments with the donor PM6 (Figure 2b). The electron mobilities (*μ*_e_) of *m*‐BAIDIC and *p*‐BAIDIC pure films were calculated to be 3.3 × 10^−4^ and 6.7 × 10^−4^ cm^2^ V^−1^ s^−1^ (Table S5, Supporting Information) using space charge limited current (SCLC) measurements^[^ ^60^ ^]^ (Figure S3, Supporting Information). Molecular packing of *m*‐BAIDIC and *p*‐BAIDIC was then investigated by grazing‐incidence wide‐angle X‐ray scattering (GIWAXS). The pure *m*‐BAIDIC film is almost amorphous (Figure S19b, Supporting Information), no obvious scattering peaks are observed even after high‐temperature annealing (180 ℃ for 10 min) (Figure 6a).^[^ ^61^ ^]^ In contrast, the pure *p*‐BAIDIC exhibits a weak lamellar peak at *q*_r_ = 0.341 Å^−1^ (*d* = 18.4 Å) (Figure S19c, Supporting Information), and the same temperature annealing could manifest a preferential face‐on packing with a lamellar peak at *q*_r_ = 0.353 Å^−1^ (*d* = 17.8 Å) and a *π*–*π* peak at *q*_z_ = 1.69 Å^−1^ (*d* = 3.72 Å) (Figure 6e). The more ordered packing of *p*‐BAIDIC is most likely due to its lower steric hindrance, consistent with the observed higher electron mobility.

**Table 1:** Basic properties of *m*‐BAIDIC and *p*‐BAIDIC

Compound	*λ*_max_ ^a)^ [nm]		*ε* [M^−1^ cm^−1^]^b)^	*E*_g_^opt^ [eV]^c)^	*E*_g_^cv^ [eV]^d)^	HOMO [eV]^e)^	LUMO [eV]^f)^
	Solution	Film					
*m*‐BAIDIC	653	671	1.1 × 10^5^	1.68	1.80	−5.67	−3.87
*p*‐BAIDIC	667	680	1.4 × 10^5^	1.66	1.78	−5.66	−3.88

^a)^ Absorption maximum;

^b)^ Molar attenuation coefficient at *λ*_max_ in solution;

^c)^ Calculated from absorption edge of thin film;

^d)^ Estimated from CV;

^e)^ Estimated from onset oxidation potential;

^f)^ Estimated from onset reduction potential.

In addition, density functional theory (DFT) calculations were employed to investigate the electronic structures of *m*‐BAIDIC and *p*‐BAIDIC. The geometry optimizations in ground states were carried out at B3LYP/6‐31G(d) level,^[^ ^62^ ^]^ and the optimal geometries of *m*‐BAIDIC and *p*‐BAIDIC are shown in Figure S5 in the Supporting Information. The calculations demonstrated that the geometry of *p*‐BAIDIC shows better planarity compared with that of *m*‐BAIDIC. Besides, the dihedral angle (19°) between thiophene ring and phenyl groups in *p*‐BAIDIC is significantly less than that (26°) in *m*‐BAIDIC. In this regard, *p*‐BAIDIC may possess a tighter stacking in the bulk phase. Frequency analysis was performed to confirm that we obtained the local minima of molecules on the potential energy surfaces. The solvent (chloroform) effect was included using the solvation model based on the density (SMD) model^[^ ^63^ ^]^ in all calculations. Note that the nonconjugated side chains were simplified to methyl groups in *m*‐BAIDIC and *p*‐BAIDIC. All calculations were carried out with Gaussian 16.^[^ ^64^ ^]^

First, the distributions and energies of the frontier molecular orbit (FMO) in *m*‐BAIDIC and *p*‐BAIDIC were calculated. The two molecules exhibited similar FMO distributions and energies. Especially, the LUMO of *m*‐BAIDIC and *p*‐BAIDIC were located on two end groups, and calculated LUMO energies are −3.08 and −3.09 eV, respectively (Figure 2c). Hence, the positional isomerization almost had no effect on the electronic structures, in agreement with CV and absorption results.

Next, the molecular electrostatic potential (ESP) plots were evaluated to gain insights into the nature of charge transfer from the donor to the acceptor in the excited state.^[^ ^65^ ^]^ The ESP distributions of *m*‐BAIDIC and *p*‐BAIDIC were mapped on their van der Waals surfaces (electron density isosurfaces of 0.001 au). The two molecules had similar ESP distributions (Figure 2d). They showed negative and positive ESP values on end groups and 2,2′‐((2Z,2′Z)‐((4,4,9,9‐tetrahexyl‐4,9‐dihydro‐s‐indaceno[1,2‐b:5,6‐b′]dithiophene‐2,7‐diyl)bis(methanylylidene))bis(3‐oxo‐2,3‐dihydro‐1H‐indene‐2,1‐diylidene))dimalononitrile (IDIC)‐based conjugated backbone, respectively, and corresponding minimal and maximum ESP values of ≈−44 and ≈34 kcal mol^−1^ were calculated. Thus, it indicates that there are charge‐transfer states in the two molecules upon electronic excitation.

Here, we assessed the carrier transport behaviors of *m*‐BAIDIC and *p*‐BAIDIC via calculating their hole (*λ*_h_) and electron (*λ*_e_) reorganization energies using the adiabatic potential energy surface method. Small reorganization energy leads to high carrier mobility as usual.^[^ ^66^ ^]^ Obviously, the calculated *λ*_h_ values of *m*‐BAIDIC and *p*‐BAIDIC were more significant than their *λ*_e_ values, indicating that their electron mobility could be higher than hole mobility (Figure S6, Supporting Information). Furthermore, compared with the *λ*_e_ value of *m*‐BAIDIC, the *λ*_e_ value of *p*‐BAIDIC was decreased by 0.078 eV, which showed a better electron‐transport property. The simulated mobilities agree with the trend observed from SCLC measurements.

We then employed time‐dependent DFT (TD‐DFT) to further investigate the excited‐state properties of *m*‐BAIDIC and *p*‐BAIDIC (Figure S7, Supporting Information). The indaceno[3,2‐*b*]dithiophene (IDT) and difluorinated 1,1‐dicyanomethylene‐3‐indanone (2FIC) groups of *m*‐BAIDIC and *p*‐BAIDIC are defined as fragment 1 (F1, blue), and *m*‐diphenylacetylene and *p*‐diphenylacetylene are defined as fragment 2 (F2, green). Electron–hole analysis showed that the two molecules have a similar distribution of photogenerated electrons, which are mainly located on F1. In this regard, 99.02% and 97.81% electrons are from F1 for *m*‐BAIDIC and *p*‐BAIDIC, respectively. However, the two molecules show different photogenerated hole distributions. Specifically, the 89.17% and 10.83% of photogenerated holes distribute on F1 and F2 of *m*‐BAIDIC, respectively. The contributions of F1 and F2 to photogenerated holes reduce and increase to 68.97% and 31.03% in *p*‐BAIDIC, respectively. The results verified the significant electron–hole separation tendency in *p*‐BAIDIC upon photoexcitation.

The charge transfer property is evaluated by three parameters, including *S*_r_ index, *t* indexes, and the account of charge transfer.^[^ ^67^ ^]^ Besides, the calculated *S*_r_ of *p*‐BAIDIC is smaller, and the *t* index of *p*‐BAIDIC is more negative than those of *m*‐BAIDIC, important indicators of charge transfer states.^[^ ^68^ ^]^ Furthermore, the account of electron transfer in *p*‐BAIDIC (0.08 e) is also higher than that of *m*‐BAIDIC (0.03 e). These results demonstrate that *p*‐BAIDIC has a more pronounced charge transfer tendency.

In addition, transfer integrals play an important role in determining carrier mobility. The transfer integral in organic semiconductors is highly sensitive to the relative orientation of adjacent molecules. Previous reports have shown that the molecular crystal structure can be predicted by using the polymorph module. Here, four possible charge carrier hopping pathways are selected on the basis of predicted crystal structures (Figure S8, Supporting Information). However, these structures are not observed in GIWAXS patterns due to the lack of long‐range order. Anyway, the calculations showed that the transfer integrals of four carriers hopping in *m*‐BAIDIC were slightly larger than those of *p*‐BAIDIC. Given that the electron mobility of *p*‐BAIDIC is higher than that of *m*‐BAIDIC, we speculate that the reorganization energies of the two molecules play a more critical role than transfer integral in determining electronic mobility here. In this regard, due to the dominant role of reorganization energy, the carrier mobility of *p*‐BAIDIC may be larger than *m*‐BAIDIC in both binary and ternary mixtures.

### 2.2 Photovoltaic Properties

**Figure 3:**
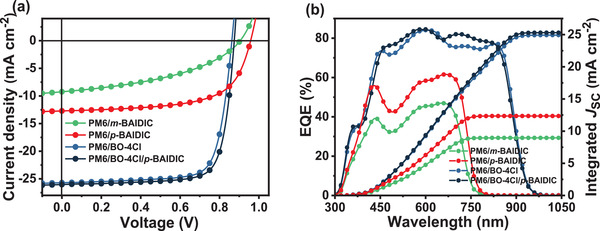
a) *J*−*V* characteristics and b) EQE spectra of the optimized devices.

The polymer donor PM6 (Figure 1) was selected to fabricate the conventional structure OSCs due to its suitable light absorption range and energy levels with the two acceptors (Figure 2). The donor/acceptor(s) weight ratio of binary and ternary OSCs was chosen to be 1/1.2 in this work. Figure S9 and Table S1 in the Supporting Information display the current density–voltage (*J*–*V*) curves and *V*_OC_, *J*_SC_, FF, and PCE values of *m*‐BAIDIC‐ and *p*‐BAIDIC‐based OSCs under different annealing temperatures (ATs), respectively. Compared to the as‐cast devices, both *m*‐BAIDIC‐ and *p*‐BAIDIC‐based devices yield higher efficiency with the AT of 100 ℃. In details, the champion *m*‐BAIDIC‐based device displays a *V*_OC_ of 0.905 V, a *J*_SC_ of 9.25 mA cm^−2^, an FF of 42.7%, and a PCE of 3.57%, whereas the best PCE of *p*‐BAIDIC‐based OSCs is 7.71% with a *V*_OC_ of 0.958 V, a *J*_SC_ of 12.7 mA cm^−2^, and an FF of 63.3% (**Figure** **3**a, **Table** **2**, and Figure S11, Supporting Information). Despite similar energy levels and absorption range, the linear *p*‐BAIDIC‐based devices exhibit much better device characteristic parameters, demonstrating that the positional isomerization has a considerable impact on the OSCs performance. The external quantum efficiency (EQE) spectra of *m*‐BAIDIC‐ and *p*‐BAIDIC‐based devices (Figure 3b, Table 2, and Figure S11, Supporting Information) display similar range but considerable differences in magnitude, and the calculated *J*_SC_ of all cells agrees with the measured values by *J*−*V* curves (< 3% mismatch).

**Table 2:** Performance of the optimized OSCs based on PM6/acceptor(s)

Acceptor(s)^a)^	*V*_OC_ ^b)^	*J*_SC_ ^b)^	FF^b)^	PCE^b)^	Calculated *J*_SC_
	[V]	[mA cm^−2^]	[%]	[%]	[mA cm^−2^]
*m*‐BAIDIC	0.905 (0.901±0.006)	9.25 (9.05±0.16)	42.7 (41.9±0.6)	3.57 (3.42±0.09)	8.95
*p*‐BAIDIC	0.958 (0.956±0.004)	12.7 (12.8±0.1)	63.3 (62.9±0.4)	7.71 (7.66±0.04)	12.4
BO‐4Cl	0.863 (0.858 ± 0.004)	25.7 (25.5 ± 0.2)	76.0 (75.0 ± 0.9)	16.9 (16.7 ± 0.2)	24.9
BO‐4Cl/*p*‐BAIDIC^c)^	0.875 (0.872 ± 0.004)	26.0 (26.0 ± 0.1)	77.2 (77.1 ± 0.1)	17.6 (17.5 ± 0.1)	25.3

^a)^ PM6/acceptor(s) = 1/1.2 w/w

^b)^ Average values (in parenthesis) are obtained from 20 devices

^c)^ 10 wt% *p*‐BAIDIC in acceptors.

Furthermore, *p*‐BAIDIC was introduced as the third component into the PM6/BO‐4Cl binary OSCs to broaden the absorption while maintaining the donor/acceptors ratio. Obviously, the values of *V*_OC_, *J*_SC_, FF, and PCE increase simultaneously when the content of *p*‐BAIDIC in acceptors increases from 0 to 10 wt%, and the ternary OSCs yield a high PCE of 17.6% with a *V*_OC_ of 0.875 V, a *J*_SC_ of 26.0 mA cm^−2^, and an FF of 77.2%. However, further increasing the concentration of *p*‐BAIDIC will have a negative impact on device performance (Table 2 and Figure S10, Table S2, and Figure S11, Supporting Information). In contrast, ternary OSCs based on *m*‐BAIDIC show no obvious improvement from the binary counterpart (Figure S12 and Table S3, Supporting Information).

The exciton dissociation and charge collection abilities are directly related to efficiency of devices. Therefore, the relationship between the photocurrent density (*J*_ph_) and the effective voltage (*V*_eff_) for the binary and ternary OSCs is plotted in Figure S13 in the Supporting Information, and the parameters of *J*_sat_, *J*_ph_^a^, *J*_ph_^b^, *η*_diss_, and *η*_coll_ are summarized in Table S4 in the Supporting Information. Here, *J*_sat_ is the saturation photocurrent density; *J*_ph_^a^ and *J*_ph_^b^ are the photocurrent density under short circuit and the maximum power output condition, respectively; *η*_diss_ and *η*_coll_ are the exciton dissociation and charge collection efficiencies, respectively; *η*_diss_ = *J*_ph_^a^/*J*_sat_; *η*_coll_ = *J*_ph_^b^/*J*_sat_. The *η*_diss_ and *η*_coll_ of the best PM6/*m*‐BAIDIC device are 70.5% and 43.9%, respectively, while those values of the corresponding *p*‐BAIDIC‐based device are 85.8% and 66.9%. In addition, the photocurrent density of the *p*‐BAIDIC‐based device reaches saturation faster than the *m*‐BAIDIC‐based device. Together, it is suggested that the *p*‐BAIDIC has more advantages than the *m*‐BAIDIC on exciton dissociation and charge collection. Consequently, the *η*_diss_ and *η*_coll_ of the optimal ternary system with *p*‐BAIDIC as a second acceptor are 95.3% and 86.9%, respectively, slightly better than the PM6/BO‐4Cl binary control (94.8% and 86.3%, respectively), demonstrating its impact in regulating ternary morphology.

The hole and electron mobilities of the binary and ternary blend films were investigated by SCLC measurements (Figure S4 and Table S5, Supporting Information). The hole mobility (*μ*_h_) and *μ*_e_ of the *p*‐BAIDIC‐based blend film are 8.5 × 10^−4^ and 1.9 × 10^−4^ cm^2^ V^−1^ s^−1^, respectively, which are twice as large as that of the *m*‐BAIDIC‐based blend film. In addition, the optimal ternary blend film exhibits relatively higher *μ*_h_ (9.3 × 10^−4^ cm^2^ V^−1^ s^−1^) and *μ*_e_ (5.9 × 10^−4^ cm^2^ V^−1^ s^−1^) with more balanced *μ*_h_/*μ*_e_ (1.6) in compared with the binary control, contributing to the observed higher FF. These results strongly support our speculation from theoretical calculations that carrier mobility of *p*‐BAIDIC is larger than *m*‐BAIDIC in both binary and ternary mixtures.

### 2.3 Energy Loss Analysis

To understand the *V*_OC_ differences of the positional isomers, the voltage loss (*V*_loss_) of the optimal OSCs has been studied. The total voltage losses can be divided into three parts according to Equation (1)^[^ ^69^ ^,^ ^70^ ^]^

(1) $$\begin{array}{ccc} qV_{\mathrm{loss}} & = & E_{g}-qV_{\mathrm{OC}}\\ & = & \left(E_{g}-qV_{\mathrm{OC},\;\mathrm{sq}}\right)+\left(qV_{\mathrm{OC},\;\mathrm{sq}}-qV_{\mathrm{OC},\;\mathrm{rad}}\right)+q\Delta V_{\mathrm{OC},\;\mathrm{nonrad}}\\ & = & q\Delta V_{1}+q\Delta V_{2}+q\Delta V_{3} \end{array}$$

where *q* is the elementary charge, *V*_OC, sq_ is the maximum voltage by the Shockley–Queisser limit. *V*_OC, rad_ is the open‐circuit voltage when there is only radiative recombination, and Δ*V*_OC, nonrad_ is the voltage loss due to nonradiative recombination, which can be determined by electroluminescence (EL) external quantum efficiencies (EQE_EL_) of the devices through Equation (2)

(2) $$\Delta V_{\mathrm{OC},\mathrm{nonrad}}=\frac{kT}{q}\;\ln\left({\mathrm{EQE}}_{\mathrm{EL}}^{-1}\right)$$

*T* is the absolute temperature in Kelvin.

**Figure 4:**
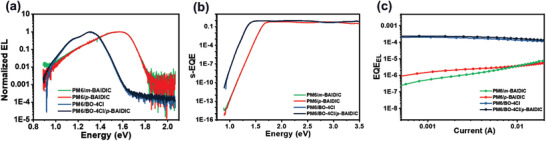
Energy loss analysis. a) Electroluminescence spectra of devices based on the blended films. b) s‐EQE and c) EQE_EL_ of the devices.

To determine the *V*_OC, rad_, we used the principle of detailed balance^[^ ^71^ ^]^ to make a connection between the low‐energy, charge‐transfer‐dominated part of the highly sensitive EQE (s‐EQE) spectra and EL spectra (**Figure** **4**a,b). Integrating the s‐EQE spectra over energy and multiplying with *q* yield the radiative emission current density that follows a diode law with the radiative saturation current density given by Equation (3)

(3) $$J_{0,\;\mathrm{rad}}\;=\;q\int_{-\infty }^{\infty }s-\mathrm{EQE}(E)\emptyset_{\mathrm{BB}}\left(E)d(E\right)$$

∅_BB_(E) denotes the blackbody spectrum at temperature *T* of the cell. Subsequently, *V*_OC, rad_ can be calculated using Equation (4)

(4) $$V_{\mathrm{OC},\;\mathrm{rad}}\;=\;\frac{\mathrm{nkT}}{q}\ln\left(\frac{J_{\mathrm{SC}}}{J_{0,\;\mathrm{rad}}}\right)$$

Here *n* is the radiative ideality factor.

Generally, the energy bandgap of the acceptors is smaller than that of donors in OSCs, thus, in this work, the *E*_g_ of the blend films is estimated from the absorption edge of neat acceptors. Then, the *V*_OC, sq_ could be directly obtained according to the Shockley–Queisser limit. The similar *E*_g_ (1.680 and 1.660 eV) indicated that devices based on *m*‐BAIDIC and *p*‐BAIDIC have similar *V*_OC, sq_ and the corresponding voltage loss (Δ*V*_1_) (**Table** **3**). Meanwhile, the devices based on *m*‐BAIDIC have a higher Δ*V*_2_ of 0.131 eV compared to the *p*‐BAIDIC‐based device (0.089 eV). Together, the radiative recombination loss (Δ*V*_r_) of *p*‐ and *m*‐BAIDIC‐based devices is 0.367 and 0.412 V, respectively. This result indicated that the alteration of molecular configuration from *m*‐ to *p*‐BAIDIC has a positive effect to reduce the radiative recombination loss in OSCs. According to Figure 4c, a larger nonradiative recombination (Δ*V*_3_) of 0.363 V for *m*‐BAIDIC‐based device is calculated based on the corresponding EQE_EL_ value (5.40 × 10^−7^), while the Δ*V*_3_ of *p*‐BAIDIC‐based device is 0.335 V. Thus, the devices based on *p*‐BAIDIC have a lower *V*_loss_ (0.702 V) than that of *m*‐BAIDIC‐based devices (0.775 V), which explains the observed higher *V*_OC_ in *p*‐BAIDIC‐based devices. The voltage loss of BO‐4Cl‐based binary and ternary systems is 0.528 and 0.516 V, respectively. Meanwhile, the ternary devices have a much lower nonradiative recombination loss than that of the binary system, this phenomenon could be attributed to the ternary blend has a lower density of trap states.^[^ ^72^ ^]^ Obviously, the lower voltage loss benefits the *V*_OC_ of devices.

**Table 3:** Detailed energy loss of optimal OSCs based on PM6/acceptor(s)

Acceptor(s)	EQE_EL_	Δ*V*_1_ [V]	Δ*V*_2_ [V]	Δ*V*_3_ [V]	*E*_g_ [eV]	*V*_loss_ [V]
*m*‐BAIDIC	5.40 × 10^−7^	0.281	0.131	0.363	1.680	0.775
*p*‐BAIDIC	1.49 × 10^−6^	0.278	0.089	0.335	1.660	0.702
BO‐4Cl	1.98 × 10^−4^	0.261	0.054	0.213	1.391	0.528
BO‐4Cl/*p*‐BAIDIC^a)^	2.31 × 10^−4^	0.261	0.046	0.209	1.391	0.516

^a)^ 10 wt% *p*‐BAIDIC in acceptors.

The carrier dynamics were performed to investigate the difference on *V*_loss_. According to the literature report before,^[^ ^73^ ^]^ the Δ*V*_r_ and Δ*V*_nr_ have a strong affiliation with the radiative recombination rate constant *k*_r_ and the nonradiative recombination rate constant *k*_nr_, which is *k*_nr_ >> *k*_r_. The affiliation is described by Equations (5) and (6) and the corresponding detailed derivation is shown in the Supporting Information

(5) $$\Delta V_{r}\propto \ln\left(k_{r}\right)$$

(6) $$\mathrm{EQ}E_{\mathrm{EL}}=\frac{k_{r}}{k_{r}+k_{\mathrm{nr}}}\;\approx \frac{k_{r}}{k_{\mathrm{nr}}}$$

The increased Δ*V*_r_ (Δ*V*_r_ = ΔV_1_   +  ΔV_2_) from 0.367 V (PM6/*p*‐BAIDIC) to 0.412 V (PM6/*m*‐BAIDIC) indicated that the *k*_r_ of the *p*‐BAIDIC‐based devices is lower than that of *m*‐BAIDIC. Meanwhile, the relatively larger EQE_EL_ and smaller *k*_r_ of the *p*‐BAIDIC‐based OSCs implies a smaller *k*_nr_ compare with *m*‐BAIDIC‐based devices. Therefore, the transient photovoltage decay measurements were performed to characterize the charge carrier lifetime which is closely related to the *k*_nr_. As shown in Figure S14a in the Supporting Information, the time constants of the OSCs based on *m*‐ and *p*‐BAIDIC are plotted as the function of the photovoltage (*V*_ph_) which was generated by the bias illumination. The decay time of *p*‐BAIDIC‐based OSCs is longer than that of the *m*‐BAIDIC under the same photovoltage, confirming that the *p*‐BAIDIC‐based devices have a lower *k*_nr_, and suggesting that Δ*V*_nr_ of the OSCs based on *p*‐BAIDIC is lower than that of *m*‐BAIDIC. For the BO‐4Cl‐based binary and ternary systems (Figure S14b, Supporting Information), the Δ*V*_r_ of the binary and ternary systems are 0.315 and 0.307 V, respectively, and the higher EQE_EL_ of the ternary devices indicated that the *k*_nr_ is lower than that of the *m*‐BAIDIC. Overall, we find that the difference on the *V*_loss_ of the OSCs in this work is mainly caused by the change of the recombination rate constant, and eventually leads to the difference of *V*_OC_.

### 2.4 Charge Transfer Kinetics

**Figure 5:**
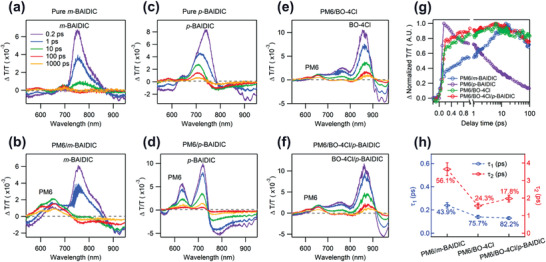
a–f) The representative fs TA spectra of pure and blend films at indicated delay times under 720 nm excitation. g) TA kinetics of blend films showing hole–electron transfer process. h) Comparisons of *τ*_1_ and *τ*_2_ of relevant blends.

The hole transfer process within the binary and ternary blends are unveiled by the fs‐transient absorptions spectroscopies (TAS). Here, a 720 nm pump pulse (≈10 µJ cm^−2^) was used to excite only acceptors without exciting the donor in blend films and after a certain delay time, the relative transmittance change (Δ*T*/*T*) was probed using a white‐light continuum. **Figure** **5**a–d and Figure S15 in the Supporting Information show a few representative TA spectra at selected delay times and 2D color plot spectra for pure *m*‐BAIDIC, PM6/*m*‐BAIDIC, pure *p*‐BAIDIC, and PM6/*p*‐BAIDIC, respectively. The bleach peak appeared at ≈700 and ≈750 nm, corresponding to the characteristic transient signals of *m*‐BAIDIC and *p*‐BAIDIC, respectively. In the TA spectra of blend films, with the decreasing of acceptor bleach peak in the first 100 ps, a new bleach from 580 to 660 nm appeared, which was assigned to the PM6 ground state bleach (GSB) signal (Figure S15, Supporting Information). Therefore, the rising kinetics of PM6 GSB in the blends directly reflects the existence of the hole transfer process, while the decay on a ps‐ns time scale corresponds to the charge recombination process.

Hole transfer was also observed in PM6/BO‐4Cl and PM6/BO‐4Cl/*p*‐BAIDIC blends from TA spectra (Figure 5e,f and Figure S16, Supporting Information). As reported before,^[^ ^74^, ^75^ ^]^ the hole transfer kinetics in OSC blends can be fitted by a biexponential function: *I* = *A*_1_ exp(−*t*/*τ*_1_) + *A*_2_ exp(−*t*/*τ*_2_), where *τ*_1_ and *τ*_2_ are the fast and slow lifetimes and *A*_1_ and *A*_2_ are the prefactors. Generally, the charge transfer process consists of an ultrafast transfer process at the interface (*A*_1_, *τ*_1_) and the diffusion‐mediated process is strongly controlled by phase segregation (*A*_2_, *τ*_2_). All the fitting parameters are presented in Figure 5h and Table S6 in the Supporting Information.

Compared with PM6/*m*‐BAIDIC, PM6/*p*‐BAIDIC shows a faster hole transfer and charge recombination process in PM6/*m*‐BAIDIC (recombination half‐lifetime ≈2 ps) presented in Figure 5g, agreed with the observed lowest charge separation efficiency (Table S4, Supporting Information) and the smallest electron mobility (Table S5, Supporting Information), leading to lower *J*_SC_, FF, and PCE in devices. Furthermore, since the rate of acceptor exciton recombination has no obvious difference for BO‐4Cl and BO‐4Cl/*p*‐BAIDIC (Figure S17, Supporting Information), the ternary blend (PM6/BO‐4Cl/*p*‐BAIDIC) showed a faster hole transfer rate than the binary one (PM6/BO‐4Cl), mainly by improving the proportion of the ultrafast hole transfer process (from 75.7% to 82.2%), suggesting the optimized donor/acceptor interface and higher hole transfer efficiency in ternary films, in good agreement with the enhancement of *J*_SC_ and PCE in ternary devices.

### 2.5 Film Morphology

**Figure 6:**
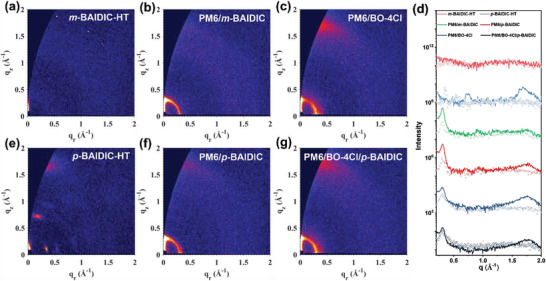
GIWAXS patterns of a) *m*‐BAIDIC‐HT, e) *p*‐BAIDIC‐HT, b) PM6/*m*‐BAIDIC, f) PM6/*p*‐BAIDIC, c) PM6/BO‐4Cl, g) PM6/BO‐4Cl/*p*‐BAIDIC films, and d) the corresponding intensity profiles along the in‐plane (dotted line) and out‐of‐plane (solid line) directions.

Atomic force microscope and transmission electron microscopy (TEM) characterizations were then used to study the surface morphology of the blend films (Figure S18, Supporting Information). Root‐mean‐square roughness of PM6/*m*‐BAIDIC, PM6/*p*‐BAIDIC, PM6/BO‐4Cl, and PM6/BO‐4Cl/*p*‐BAIDIC active layers is measured to be 1.7, 1.8, 3.0, and 3.8 nm, respectively. Both positional isomer blend films exhibited relatively smoother surfaces, whereas BO‐4Cl‐based devices show clear fibrous surface. TEM images can offer complementary local structural information about the bulk morphology of these films. Both *m*‐BAIDIC and *p*‐BAIDIC‐based binary films exhibit homogenous phase distribution. At the same time, more obvious fibrillar structures and interpenetrating networks can be seen from *p*‐BAIDIC‐based binary films, which are conducive to charge generation and extraction. Moreover, the similar fibrillar structures are observed from the ternary blends with 10 wt% *p*‐BAIDIC, which can explain the high *J*_SC_ and FF of the ternary devices. In addition, the 2D GIWAXS patterns and the intensity profiles of the pure PM6, BO‐4Cl, and blend films are displayed in Figure S19 in the Supporting Information and **Figure** **6**. The PM6 exhibits obvious face‐on orientation with a (100) lamellar peak at *q*_r_ = 0.297 Å^−1^ (*d* = 21.2 Å), and a (010) *π*–*π* peak at *q*_z_ = 1.72 Å^−1^ (*d* = 3.65 Å), and the BO‐4Cl also shows preferential face‐on orientational order with a (100) lamellar peak at *q*_r_ = 0.263 Å^−1^ (*d* = 23.9 Å), and a (010) *π*–*π* peak at *q*_z_ = 1.76 Å^−1^ (*d* = 3.57 Å). The PM6/*m*‐BAIDIC blend film shows almost no *π*–*π* peak and weak scattering intensity, indicating its low crystallinity and orientational order, whereas PM6/*p*‐BAIDIC blend film shows a relatively stronger *π*–*π* scattering peak at *q*_z_ = 1.77 Å^−1^ (*d* = 3.55 Å), indicating the retaining of face‐on stacked PM6 domains which is beneficial to vertical charge transport. Thus, the PM6/*p*‐BAIDIC‐based device exhibits higher *J*_SC_ and FF due to the stronger crystallinity and efficient charge transport pathway. In addition, the ternary film exhibits relatively tighter *π*–*π* stacking (*q*_z_ = 1.79 Å^−1^, *d* = 3.51 Å) than the BO‐4Cl‐based binary film (*q*_z_ = 1.76 Å^−1^, *d* = 3.57 Å), the tighter molecular stacking facilitates the efficient charge transport and finally improves the *J*_SC_ and FF.

## 3 Conclusion

In summary, the two positional isomers (*m*‐BAIDIC and *p*‐BAIDIC) were designed and synthesized as electron acceptors. Theoretical calculations demonstrated that *p*‐BAIDIC possesses stronger charger transfer and more planar structure than *m*‐BAIDIC. Besides, the *p*‐BAIDIC also showed smaller reorganization energy, which results in a higher carrier mobility than *m*‐BAIDIC. These properties are beneficial to improve the *J*_SC_ and reduce both radiative and nonradiative recombination‐induced energy loss. Experimental results verified that the PM6/*p*‐BAIDIC device indeed yields a much higher efficiency of 7.71% compared with the binary device of PM6/*m*‐BAIDIC. This work suggested that molecular conformation is influential to the molecular packing, electron reorganization energies, charge transport, and transfer process within OSCs, and it is worthwhile to explore appropriate isomerization strategies when designing new high‐performance organic photovoltaic materials.

## 4 Experimental Section

Details of material synthesis, device fabrication, measurement and characterization can be found in the Supporting Information.

### Statistical Analysis

Statistical analyses were performed using Origin 2018. Data presentation and sample size were exhibited as the mean ± SD in the corresponding table.

## Conflict of Interest

The authors declare no conflict of interest.

## Supporting information

Supporting Information

## Data Availability

Research data are not shared.
