# Supplementary material for: Revealing the Sole Impact of Acceptor's Molecular Conformation to Energy Loss and Device Performance of Organic Solar Cells through Positional Isomers
Source: Adv Sci (Weinh). 2022 Mar 24;9(15):2103428. doi: 10.1002/advs.202103428 (PMC9130893; doi:10.1002/advs.202103428)
Supplement: Supplementary file 1 — Supporting Information [file ADVS-9-2103428-s001.pdf]

ADVANCED SCIENCE
Open Access

Supporting Information

for *Adv. Sci.*, DOI 10.1002/advs.202103428

Revealing the Sole Impact of Acceptor's Molecular Conformation to Energy Loss and Device Performance of Organic Solar Cells through Positional Isomers

Guilong Cai, Zeng Chen, Mengyang Li, Yuhao Li, Peiyao Xue, Qingbin Cao, Weijie Chi, Heng Liu, Xinxin Xia, Qiaoshi An*, Zheng Tang*, Haiming Zhu*, Xiaowei Zhan and Xinhui Lu**

Supporting Information

Revealing the Sole Impact of Acceptor's Molecular Conformation to Energy loss and Device Performance of Organic Solar Cells Through Positional Isomers

Guilong Cai, Zeng Chen, Mengyang Li, Yuhao Li, Peiyao Xue, Qingbin Cao, Weijie Chi, Heng Liu, Xinxin Xia, Qiaoshi An,* Zheng Tang,* Haiming Zhu,* Xiaowei Zhan and Xinhui Lu**

Materials

Unless stated otherwise, all the chemical reagents and solvents used were obtained commercially and were used without further purification. Chlorobenzene (97.0%) was purchased from J&K Chemical Inc. PM6 (M_n = 20.2 kg mol^{-1}, M_w/M_n = 2.0), BO-4Cl and PDIN were purchased from eFlexPV Ltd. Compounds **2** and **3** were purchased from TCI. 2FIC[S1] and compound **1**[S2] were synthesized according to our reported procedures.

Synthesis of *m*-BAIDIC and *p*-BAIDIC

***m*-BAID-CHO.** Compound **1** (250 mg, 0.35 mmol), compound **2** (65 mg, 0.15 mmol), K_2CO_3 (420 mg, 3.0 mmol), Aliquat 336 (1 drop), and $Pd(PPh_3)_4$ (18 mg, 0.015 mmol) were dissolved in deoxygenated toluene/H_2O (20 mL, 2/1, v/v). The mixture was stirred at 110 °C for 48 h, and then cooled to room temperature. 25 mL of water was added and the mixture was extracted with dichloromethane (2 × 50 mL). The organic phase was dried over anhydrous $MgSO_4$ and filtered. After removal of

the solvent under reduced pressure, the residue was purified by column chromatography on silica gel using a mixture solvent as eluent (petroleum ether/dichloromethane, v/v = 1/2) to give a yellow solid (174 mg, 81%). ^{1}H NMR (400 MHz, $CDCl_3$): δ 9.83 (s, 2H), 7.87 (s, 2H), 7.69–7.58 (m, 4H), 7.45 (s, 4H), 7.37 (m, 4H), 7.31 (s, 2H), 2.18–1.72 (m, 16H), 1.39–0.94 (m, 64H), 0.82 (m, 24H). ^{13}C NMR (100 MHz, CD_2Cl_2): δ 183.3, 158.0, 155.9, 155.7, 154.1, 152.7, 146.6, 145.4, 141.7, 139.0, 136.1, 134.9, 131.7, 131.2, 131.0, 129.8, 129.4, 128.9, 126.0, 124.4, 119.1, 115.5, 114.0, 90.1, 78.2, 55.0, 54.8, 39.7, 39.7, 32.2, 32.2, 30.3, 30.3, 30.2, 24.8, 23.2, 23.2, 14.4. MS (MALDI-TOF): *m/z* 1435.71 (M^+).

***p*-BAID-CHO.** Compound *p*-BAID-CHO was synthesized according to the synthetic procedure of compound *m*-BAID-CHO. Compound *p*-BAID-CHO was obtained as a blue solid (181 mg, 84%). ^{1}H NMR (400 MHz, CD_2Cl_2): δ 9.9 (s, 2H), 7.7 (m, 4H), 7.7 (s, 2H), 7.6 (m, 4H), 7.5 (s, 2H), 7.4 (s, 2H), 7.4 (s, 2H), 2.2–1.9 (m, 16H), 1.4–1.1 (m, 64H), 0.9–0.8 (m, 24H). ^{13}C NMR (100 MHz, CD_2Cl_2): δ 183.3, 158.1, 155.9, 155.7, 154.1, 152.6, 146.9, 146.9, 145.4, 141.9, 138.9, 135.6, 134.9, 133.2, 132.7, 132.2, 131.2, 128.3, 126.8, 125.7, 122.5, 120.9, 119.1, 115.5, 114.0, 91.2, 55.0, 54.8, 39.7, 39.7, 32.2, 32.2, 30.3, 30.2, 24.8, 23.2, 23.1, 14.4. MS (MALDI-TOF): *m/z* 1435.64 (M^+).

***m*-BAIDIC.** To a three-necked round bottom flask were added *m*-BAID-CHO (144 mg, 0.1 mmol), 2FIC (83 mg, 0.35 mmol), pyridine (0.15 mL) and chloroform (20 mL). The mixture was deoxygenated with nitrogen for 20 min and then stirred at reflux for 12 h. After cooled to room temperature, the mixture was poured into

methanol (200 mL) and filtered. The residue was purified by column chromatography on silica gel using a mixture solvent as eluent (petroleum ether/dichloromethane, v/v = 1/1) to give a blue solid (158 mg, 85%). ^{1}H NMR (700 MHz, CD_2Cl_2): δ 8.96 (s, 2H), 8.53 (m, 2H), 7.93 (m, 2H), 7.77 (s, 2H), 7.72 – 7.68 (m, 4H), 7.66 (s, 2H), 7.53 (d, J = 7.5 Hz, 2H), 7.46 (t, J = 7.6 Hz, 2H), 7.43 (s, 2H), 7.39 (s, 2H), 2.12 – 1.97 (m, 16H), 1.30 – 1.11 (m, 64H), 0.83 – 0.76 (m, 24H). ^{13}C NMR (175 MHz, CD_2Cl_2): δ 186.5, 164.6, 159.3, 159.3, 157.5, 157.3, 154.6, 147.9, 141.5, 141.2, 140.4, 139.3, 139.1, 135.9, 134.8, 131.2, 130.4, 129.8, 129.0, 128.7, 126.1, 124.4, 120.3, 119.1, 116.7, 115.4, 115.4, 115.3, 115.2, 114.1, 112.9, 112.8, 89.9, 68.8, 55.0, 54.8, 39.8, 39.6, 32.2, 32.1, 30.2, 30.1, 24.8, 24.8, 23.2, 23.1, 23.1, 14.4, 14.3, 14.3. MS (MALDI-TOF): *m/z* 1859.57 (M^+).

***p*-BAIDIC.** Compound *p*-BAIDIC was synthesized according to the synthetic procedure of compound *m*-BAIDIC. Compound *p*-BAIDIC was obtained as a blue solid (162 mg, 87%). ^{1}H NMR (700 MHz, CD_2Cl_2): δ 8.96 (s, 2H), 8.52 (m, 2H), 7.77 (s, 2H), 7.72 (m, 4H), 7.69 (t, J = 7.5 Hz, 2H), 7.65 (s, 2H), 7.60 (m, 4H), 7.43 (s, 2H), 7.37 (s, 2H), 2.11 – 1.95 (m, 16H), 1.33 – 1.10 (m, 64H), 0.78 (m, 24H). ^{13}C NMR (175 MHz, CD_2Cl_2): δ 186.5, 164.5, 159.5, 159.3, 157.5, 157.3, 154.7, 148.2, 141.7, 141.2, 140.5, 139.3, 139.1, 135.4, 134.8, 132.7, 130.4, 129.5, 128.7, 125.8, 122.8, 120.3, 119.1, 116.7, 115.4, 115.4, 115.3, 115.2, 114.1, 112.9, 112.8, 91.2, 68.8, 55.0, 54.8, 39.8, 39.6, 32.1, 32.1, 30.2, 30.1, 24.8, 24.8, 23.2, 23.1, 23.1, 14.4, 14.3, 14.3. MS (MALDI-TOF): *m/z* 1859.94 (M^+).

Characterization

The ^{1}H NMR and ^{13}C NMR spectra were measured by Bruker AVANCE 400 or 700 MHz spectrometer. Mass spectra were performed by Bruker Daltonics Biflex III MALDI-TOF Analyzer in the MALDI mode. The ultraviolet-visible light (UV-vis) absorption spectra were measured using the JASCO-570 spectrophotometer (JASCO. Inc., Japan) in solution (chloroform) and the thin film (on a quartz substrate). Electrochemical measurements were carried out under nitrogen in a solution of tetra-*n*-butylammonium hexafluorophosphate ($[^{n}Bu_4N]^+[PF_6]^-$) (0.1 M) in CH_3CN employing a computer-controlled CHI660C electrochemical workstation, glassy carbon working electrode coated with films, an Ag/AgCl reference electrode, and a platinum-wire auxiliary electrode. The HOMO/LUMO were estimated from onset oxidation and reduction potentials referenced to a ferrocenium/ferrocene ($FeCp_2^{+/0}$) couple using ferrocene as an external standard. The active layer surface was measured in ambient conditions via an Atomic force microscope (AFM) (Bruker Dimension XR, United States) using a back side Al 100 nm coating tip (Bruker: OTESPA-R3; k: 26 N/m; f_0: 300 kHz). The scan area and speed were 2.0 μm × 2.0 μm and 0.7 Hz.

Device Fabrication and Characterization

All the devices are based on a conventional sandwich structure, the patterned indium-tin oxide glass (ITO glass)/PEDOT:PSS/active layer/PDIN/Ag. The patterned ITO glass coated substrates were consecutively cleaned in ultrasonic baths containing detergent, de-ionized water and ethanol, respectively. Then, poly-(3,4-ethylenedioxythiophene):poly-(styrenesulphonicacid) (PEDOT:PSS) thin

films were fabricated on the cleaned ITO substrates by spin-coating method at 5000 round per minute (RPM) for 40 s, and then annealed at 150 °C for 10 minutes in ambient conditions. After annealing treatment, the ITO substrates coated PEDOT:PSS films were transferred to a high-purity nitrogen-filled glove box to fabricate active layers. The polymer donor PM6 and small molecular acceptor(s) were dissolved in chlorobenzene to prepare 22 mg mL^{-1} blend solutions. The weight ratio of donor to acceptor is kept constant as 1/1.2. The blend solutions were spin-coated on PEDOT:PSS films in a high purity nitrogen-filled glove box to fabricate the active layers. The optimized thickness of the active layer is ~100 nm, which was measured by KLA-Tencor Alpha-Step D-600 Stylus Profiler. After that, PDIN solution (2 mg mL^{-1} in methanol with 0.25 vol% acetic acid) was spin-coated on the top of active layers at 5000 RPM for 30 s. The cathode of Ag was deposited by thermal evaporation with a shadow mask under 10^{-4} Pa and the thickness of 100 nm was monitored by a quartz crystal microbalance. The *J-V* curves was measured using a computer-controlled B2912A Precision Source/Measure Unit (Agilent Technologies, United States). An XES-70S1 (SAN-EI Electric Co., Ltd., Japan) solar simulator (AAA grade, 70 mm × 70 mm) coupled with AM 1.5G solar spectrum filters was used as the light source, and the optical power at the sample was 100 mW cm^{-2}. The measured area of the active device was 4 mm^2. A 2 cm × 2 cm monocrystalline silicon reference cell (SRC-1000-TC-QZ) was purchased from VLSI Standards Inc. The external quantum efficiency (EQE) spectra were measured using a Solar Cell Spectral Response Measurement System QE-R3011 (Enlitech Co., Ltd.). The light intensity at

each wavelength was calibrated through standard single crystal Si photovoltaic cell.

SCLC Measurements

Hole-only or electron-only devices were fabricated as follows: ITO/PEDOT:PSS/active layer/Au for holes and ITO/ZnO/active layer/Ca/Al for electrons. The mobility was extracted by fitting the *J*-*V* curves using space charge limited current (SCLC) method,[S3] which follows $J = (9/8)\mu\varepsilon_r\varepsilon_0V^2\exp(0.89(V/E_0d)^{0.5})/d^3$.

Here, J refers to the current density, μ is hole or electron mobility, ε_r is relative dielectric constant of the transport medium, which is equal to 3, ε_0 is the permittivity of free space (8.85×10^{-12} F m^{-1}), $V = V_{appl} - V_{bi}$, where V_{appl} is the applied voltage to the device, and V_{bi} is the built-in voltage due to the difference in work function of the two electrodes (for hole-only diodes, V_{bi} is 0.2 V; for electron-only diodes, V_{bi} is 0 V). E_0 is characteristic field, d is the thickness of the active layer and was measured by KLA-Tencor Alpha-Step D-600 Stylus Profiler.

EL Measurement

EL measurement was conducted by direct-current meter (PWS2326, Tectronix) to provide bias voltage for the test device, and the EL spectra were recorded by the fluorescence spectrometer (KYMERA-328I-B2, Andor technology LTD) with cooled silicon array and indium gallium arsenic detector, which was calibrated by standard light source (Ocean Optics).

EQE_{EL} Measurement

The EQE_{EL} was recorded with a built-in-house system comprising a standard

silicon photodiode (S1337-1010BR, Hamamatsu Electronics), Keithley 2400 source meter (for supplying voltages and recording injected currents), and Keithley 6482 picoammeter (for measuring the emitted light intensity).

s-EQE Measurement

A 150 W quartz halogen lamp (LSH-75, Newport) acted as a light source, passing through the monochromator (CS260-RG-3-MC-A, Newport) to provide an adjustable monochromatic light source for testing, and then emited an optical signal at a 173 Hz frequency through the chopper (3502 Optical Chopper, Newport) and focused on the OSC devices. The current generated by the device was amplified by the front-end current amplifier (SR570, Stanford) to reduce the impact of the noise signal. The final signal was collected and analyzed by a Phase-locked Amplifier (SR830 DSP Lock-In Amplifier, Stanford).

Transient photovoltage (TPV) decay measurement

For TPV measurements, we use an LED connected to a Keithley 2400 as a background light source, while we use another LED as a pulsed light source. The pulsed light is controlled by an arbitrary wave generator (Tektronix, AFG3022C), and the final signal acquisition and processing is performed by an oscilloscope (Tektronix, MDO4104C).

The theory of V_{loss} in OSCs

Under the open-circuit condition, the generation (G) rate and recombination (R) rate of charge carriers must be balanced (G = R). Assuming that the recombination of charge carriers takes place via CT states:

$$G = kN_{CT} \tag{1}$$

where k is the total recombination rate constant for the CT state decay, which is the sum of the radiative and the non-radiative recombination rate.[S4]

$$k = k_r + k_{nr} \approx k_{nr} \tag{2}$$

N_{CT} is the density of populated CT states, which can be expressed as:[S5]

$$N_{CT} = N_{CTC} exp\left(\frac{-E_{CT} + qV_{OC}}{k_B T}\right) \tag{3}$$

where N_{CTC} is the density of CT state complex.

The equation (3) could be reformulated, we then get the relation for the V_{OC}:

$$qV_{oc} = E_{CT} + k_B T \ln\left(\frac{G}{kN_{CTC}}\right) \tag{4}$$

We identify the second term on the right side of Equation (4) to the overall voltage losses (ΔV_{OC})

$$\Delta V_{OC} = \frac{E_{CT}}{q} - V_{OC} = \frac{k_B T}{q} \ln\left(\frac{kN_{CTC}}{G}\right) \tag{5}$$

Using equation (2) in equation (5), we derive

$$\begin{aligned} \Delta V_{OC} &= \frac{k_B T}{q} ln\left(\frac{(k_r + k_{nr})N_{CTC}}{G}\right) \\ &= \frac{k_B T}{q} \ln\left(\frac{k_r}{k_r}\frac{(k_r + k_{nr})N_{CTC}}{G}\right) \\ &= \frac{k_B T}{q} \ln\left(\frac{k_r + k_{nr}}{k_r}\right) + \frac{k_B T}{q} \ln\left(\frac{k_r N_{CTC}}{G}\right) \\ &= \Delta V_{nr} + \Delta V_r \end{aligned} \tag{6}$$

ΔVr is the radiative voltage losses and ΔV_{nr} is the non-radiative voltage losses.

$$\Delta V_r \propto \ln(k_r) \tag{7}$$

Now, ΔV_r could be divided in two parts:[S6] ΔV_1 and ΔV_2

$$\Delta V_r = \Delta V_1 + \Delta V_2 \quad (8)$$

Where, ΔV_1 is the inevitable part of the radiative recombination voltage loss, derived by the SQ, assuming that the absorption spectrum of the solar cell is a step-like function. ΔV_2 is the additional radiative voltage loss due to the fact that the real absorption spectrum of the solar cell is not a step-like function.

And

$$\Delta V_{nr} = \frac{kT}{q} \ln\left(\frac{1}{EQE_{EL}}\right) \quad (9)$$

Because

$$EQE_{EL} = \frac{k_r}{k_r + k_{nr}} \approx \frac{k_r}{k_{nr}} \quad (10)$$

Transient Absorption Spectroscopy (TAS) Measurement

For femtosecond transient absorption spectroscopy, the fundamental output from Yb:KGW laser (1030 nm, 220 fs Gaussian fit, 100 kHz, Light Conversion Ltd) was separated to two light beam. One was introduced to NOPA (ORPHEUS-N, Light Conversion Ltd) to produce a certain wavelength for pump beam (here we use 720 nm), the other was focused onto a YAG plate to generate white light continuum as probe beam. The pump and probe overlapped on the sample at a small angle less than 10°. The transmitted probe light from sample was collected by a linear CCD array. Then we obtained transient differential transmission signals by equation shown below:

$$\frac{\Delta T}{T} = \frac{T_{pump\text{-}on} - T_{pump\text{-}off}}{T_{pump\text{-}off}}$$

All the samples were measured in vacuum environments.

GIWAXS Measurements

GIWAXS measurement were accomplished with a Xeuss 2.0 WAXS/SAXS laboratory beamline using a Cu X-ray source (8.05 keV, 1.54 Å) and a Pilatus3R 300K detector. GIWAXS samples were prepared on silicon substrate by spin coating.

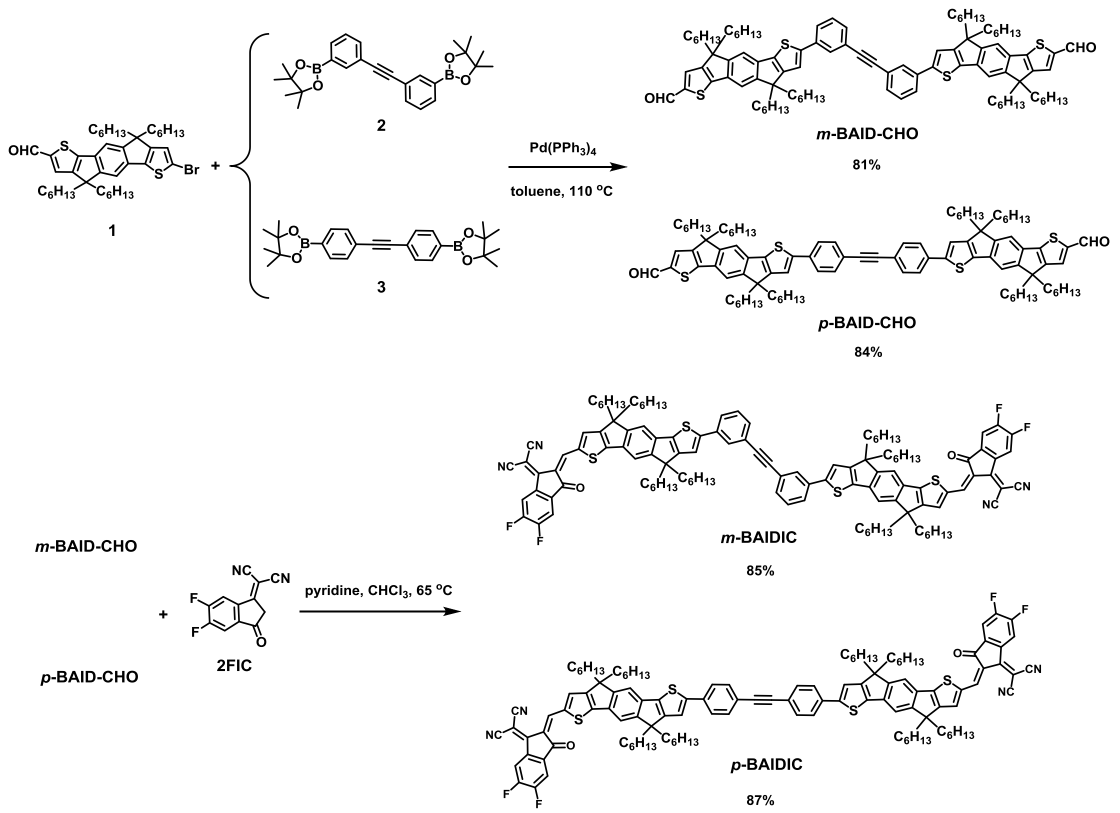

Scheme S1. Synthetic routes to *m*-BAIDIC and *p*-BAIDIC.

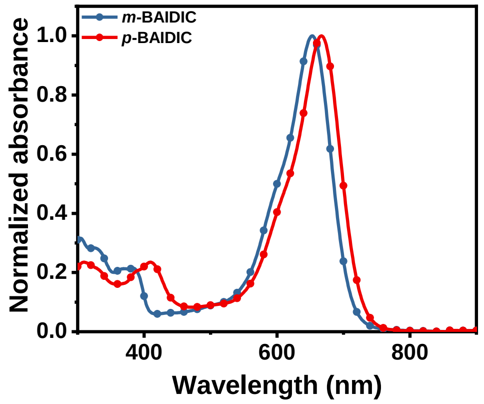

Figure S1. Absorption spectra of *m*-BAIDIC and *p*-BAIDIC in chloroform solution.

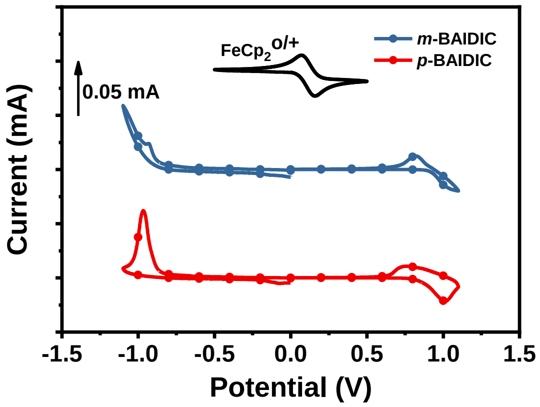

Figure S2. Cyclic voltammograms for *m*-BAIDIC and *p*-BAIDIC in CH_3CN/0.1 M Bu_4NPF_6 at 100 mV s^{-1}; the horizontal scale refers to an Ag/AgCl electrode as a reference electrode.

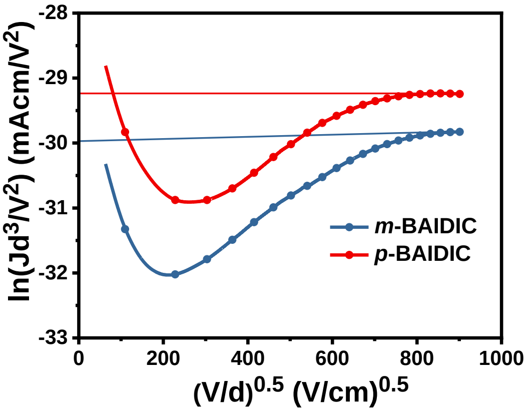

Figure S3. *J*-*V* characteristics in the dark for electron-only devices based on *m*-BAIDIC and *p*-BAIDIC.

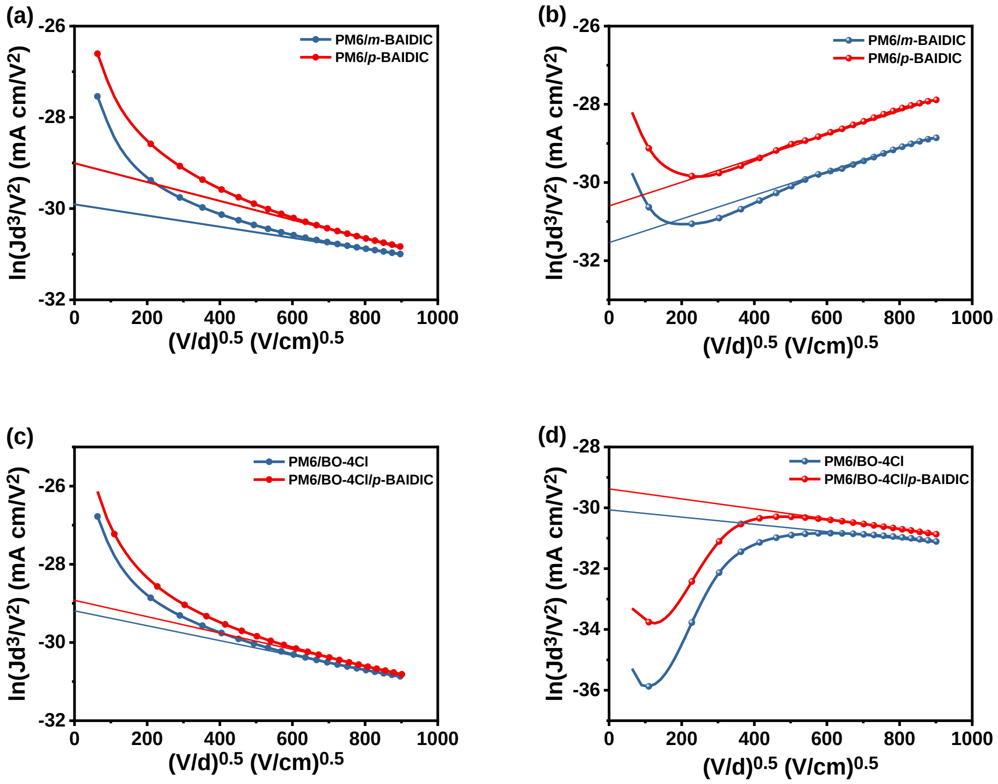

Figure S4. *J*-*V* characteristics in the dark for (a) (c) hole-only and (b) (d) electron-only devices based on optimal binary and ternary blends.

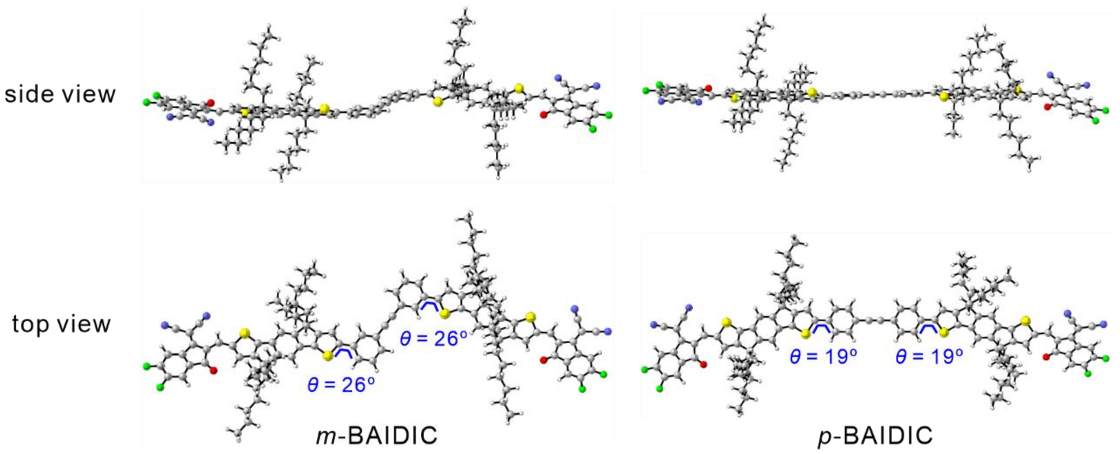

Figure **S5**. The side and top view of *m*-BAIDIC and *p*-BAIDIC.

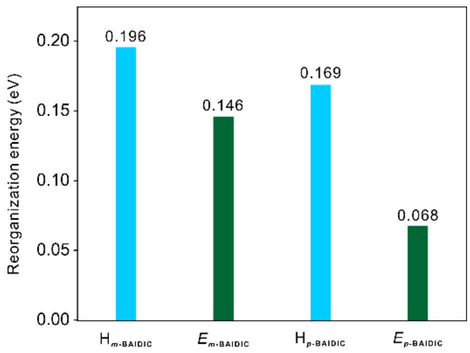

Figure S6. The hole and electron reorganization energies of *m*-BAIDIC and *p*-BAIDIC.

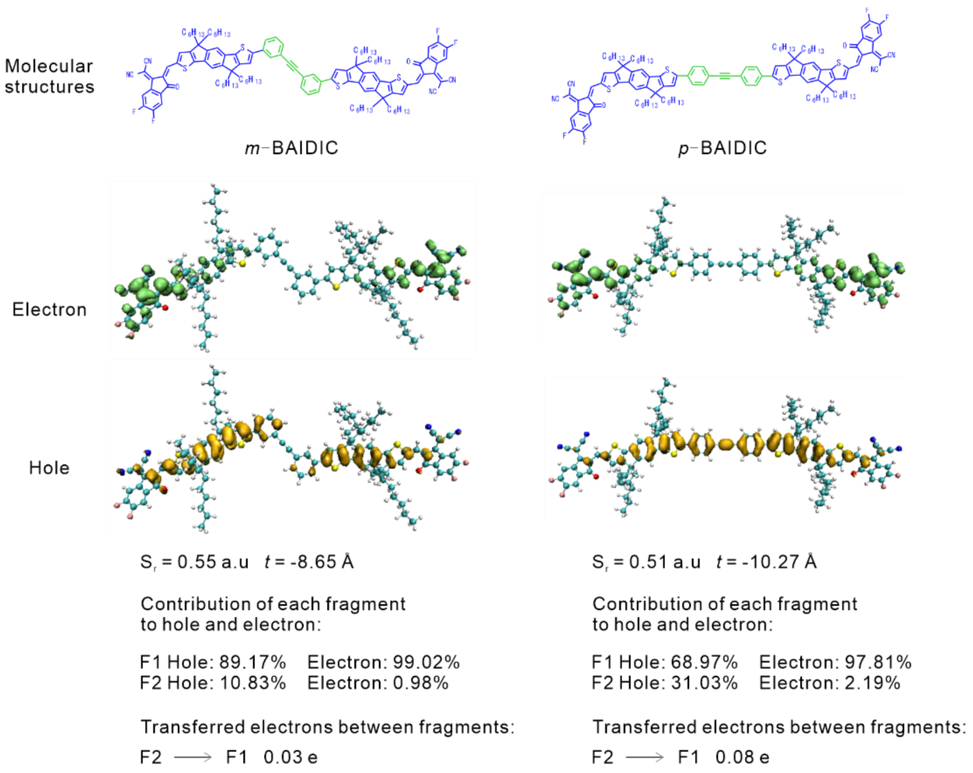

Figure S7. Calculated electron-hole distributions, S_r index, t index, and account of electron transfer in the first excited state of *m*-BAIDIC and *p*-BAIDIC.

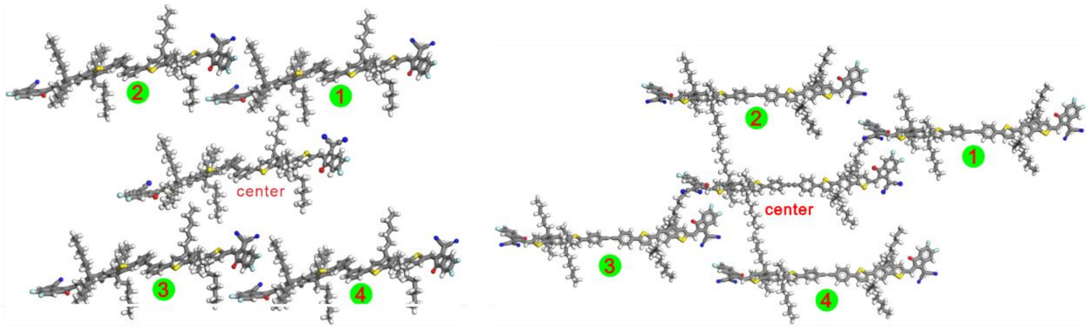

m-BAIDIC		
	V_{DA} (eV)	*r* (cm)
1	0.004	2.40×10^{-7}
2	0.004	2.69×10^{-7}
3	0.004	2.40×10^{-7}
4	0.004	2.69×10^{-7}

p-BAIDIC		
	V_{DA} (eV)	*r* (cm)
1	0.002	4.05×10^{-7}
2	0.001	2.50×10^{-7}
3	0.002	4.05×10^{-7}
4	0.001	2.50×10^{-7}

Figure S8. The selected four electron carrier transport pathways along with *m*-BAIDIC and *p*-BAIDIC transfer integrals and center of mass distances (*r*, cm).

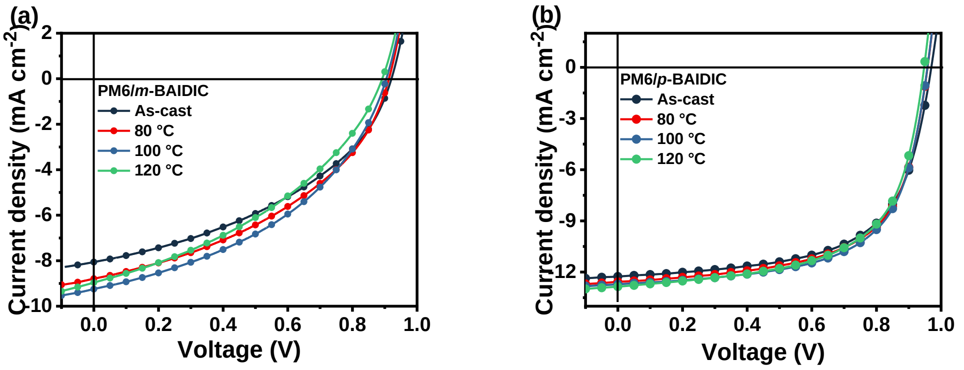

Figure S9. *J*-*V* characteristics of (a) PM6/*m*-BAIDIC and (b) PM6/*p*-BAIDIC OSCs with different annealing temperature.

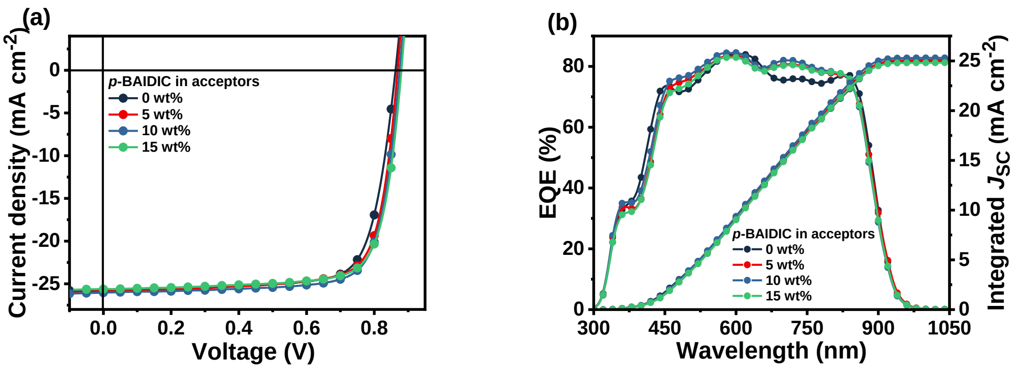

Figure S10. (a) *J-V* characteristics and (b) EQE spectra of the PM6/BO-4Cl/*p*-BAIDIC OSCs.

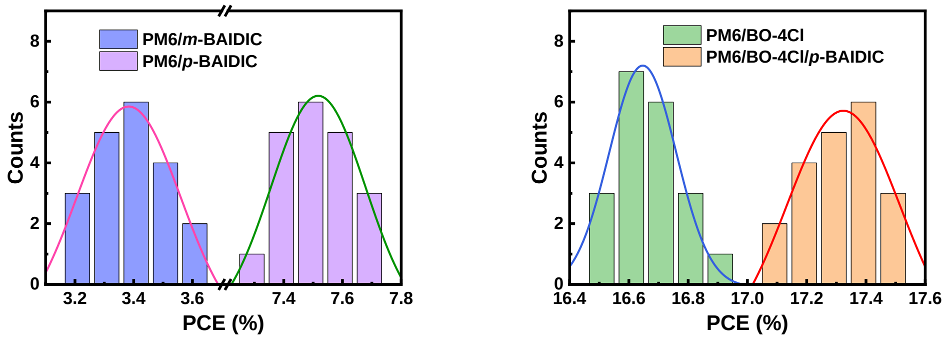

Figure S11. The PCE distribution of OSCs based on *m*-BAIDIC, *p*-BAIDIC, BO-4Cl and BO-4Cl/*p*-BAIDIC.

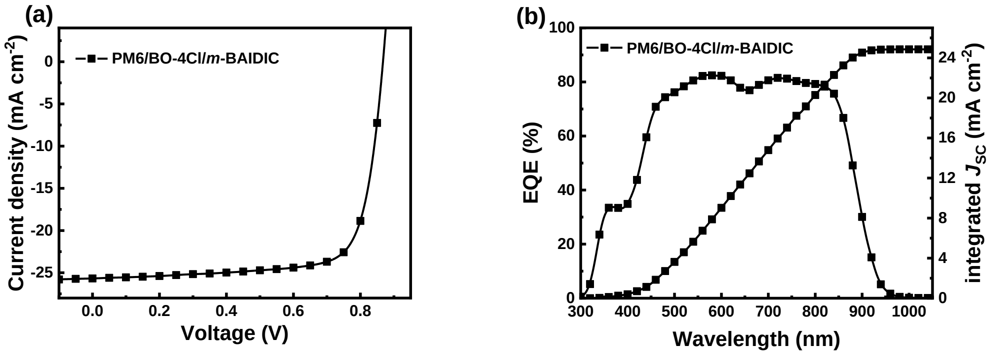

Figure S12. (a) *J*−*V* characteristics and (b) EQE spectra of the optimized devices.

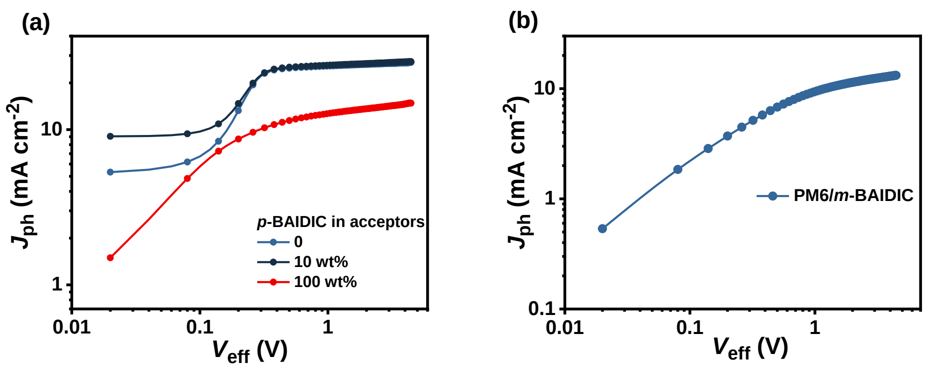

Figure S13. J_{ph} versus V_{eff} characteristics of the optimized (a) PM6/BO-4Cl/*p*-BAIDIC and (b) PM6/*m*-BAIDIC OSCs.

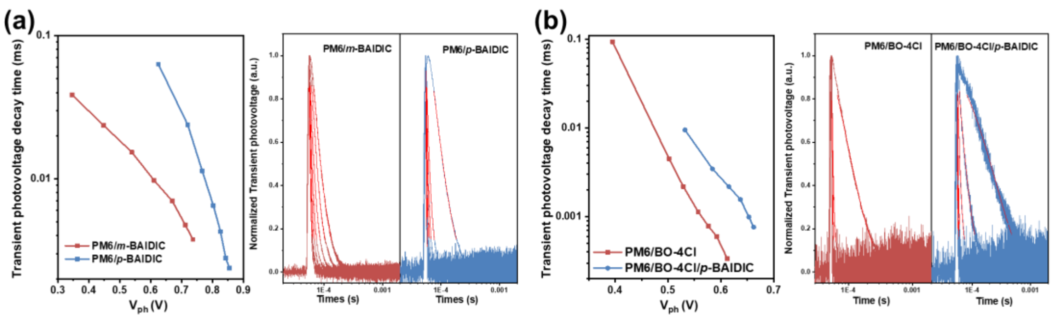

Figure S14. The results of the TPV measurement based on (a) PM6/*m*-BAIDIC, PM6/*p*-BAIDIC, (b) PM6/BO-4Cl and PM6/BO-4Cl/*p*-BAIDIC.

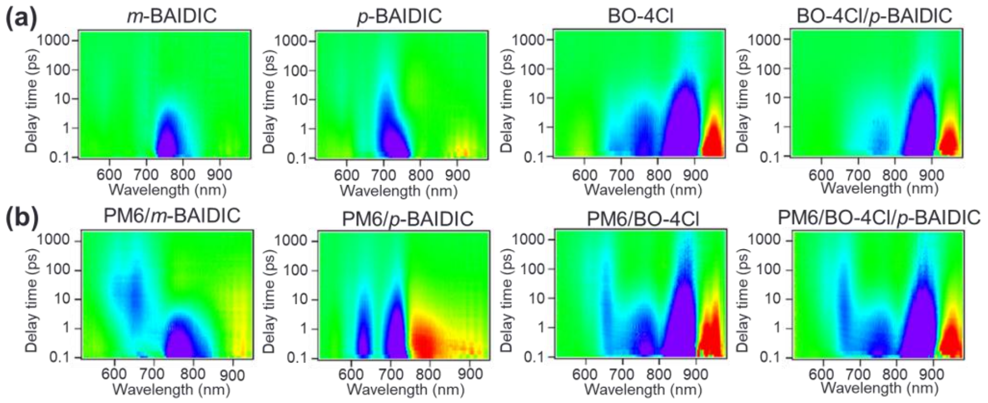

Figure S15. (a) Color plot of TA spectra of *m*-BAIDIC, *p*-BAIDIC, BO-4Cl, BO-4Cl/*p*-BAIDIC, (b) optimal PM6/*m*-BAIDIC, PM6/*p*-BAIDIC, PM6/BO-4Cl and PM6/BO-4Cl/*p*-BAIDIC (10 wt% *p*-BAIDIC in acceptors) films under 720 nm excitation.

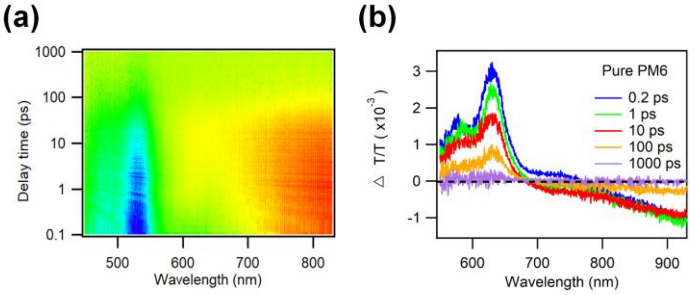

Figure S16. (a) Color plot of TA spectra of PM6 under 550 nm excitation. (b) Representative TA spectra at indicated delay time.

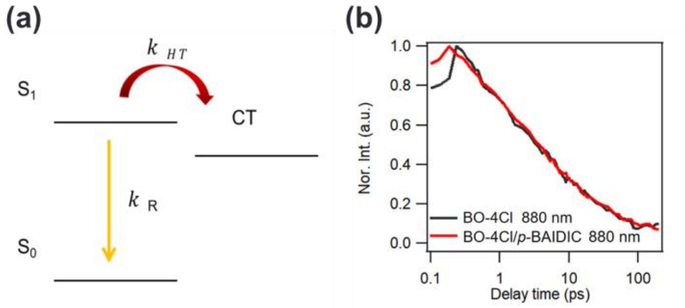

Figure S17. (a) Schematic depiction of charge generation. (b) Recombination kinetics of BO-4Cl and BO-4Cl/*p*-BAIDIC.

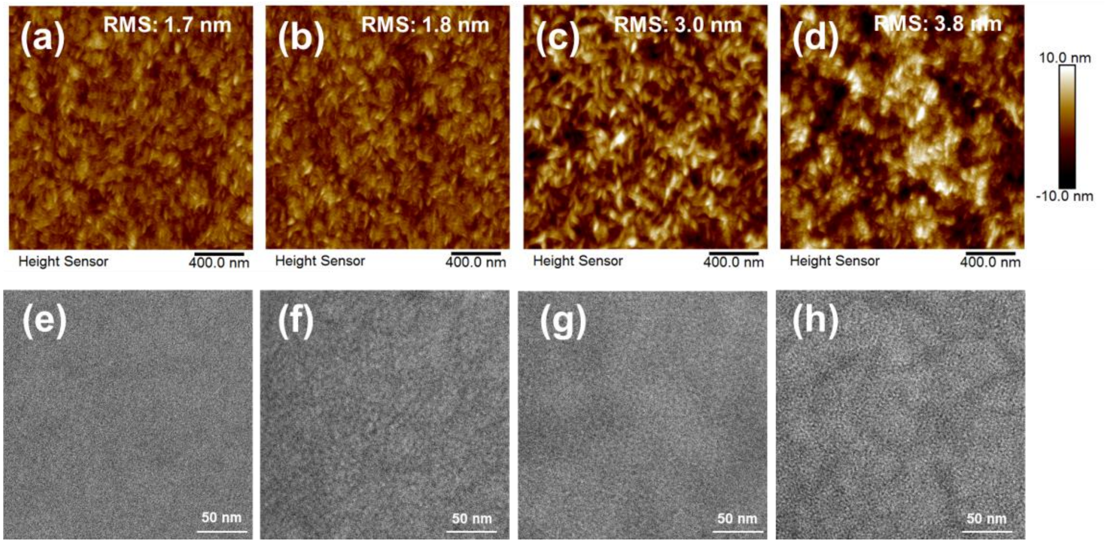

Figure S18. AFM height and TEM images of (a), (e) PM6/*m*-BAIDIC, (b), (f) PM6/*p*-BAIDIC, (c), (g) PM6/BO-4Cl and (d), (h) PM6/BO-4Cl/*p*-BAIDIC (10 wt% *p*-BAIDIC in acceptors) blend films under optimal device conditions.

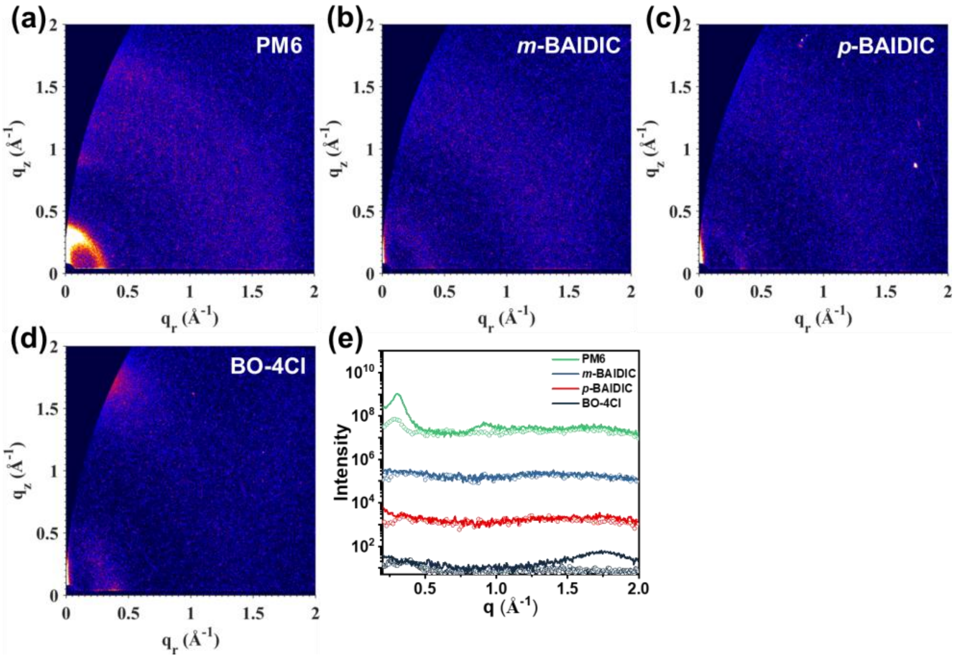

Figure S19. 2D GIWAXS patterns of (a) PM6, (b) *m*-BAIDIC, (c) *p*-BAIDIC and (d) BO-4Cl pure films; (e) the corresponding intensity profiles along the in-plane (dotted line) and out-of-plane (solid line) directions.

Table S1. Device data of OSCs based on PM6/acceptor with different AT.

acceptor	AT (℃)	V_{OC}[a] (V)	J_{SC}[a] (mA cm^{-2})	FF[a] (%)	PCE[a] (%)
m-BAIDIC	As-cast	0.921<br>(0.923±0.003)	8.06<br>(7.82±0.19)	42.0<br>(43.1±1.8)	3.11<br>(2.99±0.21)
	80	0.912<br>(0.909±0.003)	8.79<br>(8.68±0.13)	42.0<br>(40.6±1.4)	3.37<br>(3.20±0.17)
	100	0.905<br>(0.901±0.006)	9.25<br>(9.05±0.16)	42.7<br>（41.9±0.6）	3.57<br>(3.42±0.09)
	120	0.892<br>(0.890±0.003)	8.96<br>(8.89±0.14)	39.0<br>(38.1±0.9)	3.12<br>(3.01±0.09)
p-BAIDIC	As-cast	0.969<br>(0.966±0.003)	12.3<br>(11.9±0.3)	62.2<br>(61.3±1.0)	7.39<br>(7.21±0.18)
	80	0.960<br>(0.964±0.004)	12.6<br>(12.0±0.4)	62.6<br>(62.1±0.7)	7.55<br>(7.45±0.14)
	100	0.958<br>(0.956±0.004)	12.7<br>(12.8±0.1)	63.3<br>（62.9±0.4）	7.71<br>(7.66±0.04)
	120	0.948<br>(0.945±0.003)	12.9<br>(12.7±0.2)	61.6<br>(60.9±0.7)	7.50<br>(7.46±0.06)

[a] Average values (in parenthesis) are obtained from 20 devices.

Table S2. Performance of the PM6/BO-4Cl/*p*-BAIDIC OSCs with various *p*-BAIDIC content in acceptors.

p-BAIDIC in acceptors (wt%) [a]	V_{OC} [b] (V)	J_{SC} [b] (mA cm^{-2})	FF [b] (%)	PCE [b] (%)	calculated J_{SC} (mA cm^{-2})
0	0.863 (0.858±0.004)	25.7 (25.5±0.2)	76.0 (75.0±0.9)	16.9 (16.7±0.2)	24.9
5	0.869 (0.864±0.005)	25.9 (25.7±0.1)	76.4 (76.0±0.4)	17.2 (16.8±0.4)	25.1
10	0.875 (0.872±0.004)	26.0 (26.0±0.1)	77.2 (76.5±0.5)	17.6 (17.2±0.5)	25.3
15	0.880 (0.876±0.003)	25.6 (25.4±0.2)	77.0 (76.3±0.7)	17.4 (17.2±0.2)	24.8

[a] PM6/acceptor = 1/1.2 (w/w). [b] Average values (in parenthesis) are obtained from 20 devices.

Table S3. Performance of the optimized OSCs based on PM6/acceptors.

acceptors [a]	V_{OC} [b] (V)	J_{SC} [b] (mA cm^{-2})	FF [b] (%)	PCE [b] (%)	calculated J_{SC} (mA cm^{-2})
BO-4Cl/*m*-BAIDIC [c]	0.868 (0.864±0.004)	25.6 (25.5±0.2)	76.1 (76.0±0.1)	16.9 (16.8±0.1)	24.9

[a] PM6/acceptors = 1/1.2 (w/w). [b] Average values (in parenthesis) are obtained from 20 devices. [c] 10 wt% *m*-BAIDIC in acceptors.

Table S4. The parameters of exciton dissociation efficiency and charge collection efficiency.

sample	J_{sat} (mA cm^{-2})	J_{ph}^{a} (mA cm^{-2})	J_{ph}^{b} (mA cm^{-2})	η_{diss} (%)	η_{coll} (%)
PM6/*m*-BAIDIC	13.2	9.3	5.8	70.5	43.9
PM6/*p*-BAIDIC	14.8	12.7	9.9	85.8	66.9
PM6/BO-4Cl	27.1	25.7	23.4	94.8	86.3
PM6/BO-4Cl/*p*-BAIDIC[c]	27.4	26.1	23.8	95.3	86.9

[a] Under short circuit condition; [b] Under the maximal power output condition; [c] 10 wt% *p*-BAIDIC in acceptors.

Table S5. Charge Mobilities of Pure and Blend Films Measured by SCLC Method.

Active layer	μ_h (cm^2 V^{-1} s^{-1})	μ_e (cm^2 V^{-1} s^{-1})	μ_h/μ_e
m-BAIDIC	-	3.3×10^{-4}	-
p-BAIDIC	-	6.7×10^{-4}	-
PM6/*m*-BAIDIC	4.4×10^{-4}	7.0×10^{-5}	6.3
PM6/*p*-BAIDIC	8.5×10^{-4}	1.9×10^{-4}	4.5
PM6/BO-4Cl	6.8×10^{-4}	3.2×10^{-4}	2.1
PM6/BO-4Cl/*p*-BAIDIC	9.3×10^{-4}	5.9×10^{-4}	1.6

Table S6. Detailed fitting parameters of optimal OSCs based on PM6/acceptor(s).

acceptor(s)	A_1	τ_1 (ps)	A_2	τ_2 (ps)	T (ps) [a]
m-BAIDIC	0.4388	0.24 ± 0.02	0.5612	3.65 ± 0.37	2.15 ± 0.22
p-BAIDIC	~1	<100 fs	-	-	<100 fs
BO-4Cl	0.7574	0.14 ± 0.01	0.2426	1.56 ± 0.16	0.48 ± 0.05
BO-4Cl/*p*-BAIDIC [b]	0.8219	0.13 ± 0.02	0.1781	1.97 ± 0.20	0.46 ± 0.05

[a] $T = A_1\tau_1 + A_2\tau_2$. [b] 10 wt% *p*-BAIDIC in acceptors.

References

[S1] S. Dai, F. Zhao, Q. Zhang, T. K. Lau, T. Li, K. Liu, Q. Ling, C. Wang, X. Lu, W. You, X. Zhan, *J. Am. Chem. Soc.* **2017**, *139*, 1336.

[S2] G. Cai, Y. Xiao, M. Li, J. J. Rech, J. Wang, K. Liu, X. Lu, Z. Tang, J. Lian, P. Zeng, Y. Wang, W. You, X. Zhan, *J. Mater. Chem. A* **2020**, *8*, 13735.

[S3] G. G. Malliaras, J. R. Salem, P. J. Brock, C. Scott, *Phys. Rev. B* **1998**, *58*, 13411.

[S4] K. Vandewal, K. Tvingstedt, A. Gadisa, O. Inganäs, J. V. Manca, *Phys. Rev. B* **2010**, 81, 125204.

[S5] J. Benduhn, K. Tvingstedt, F. Piersimoni, S. Ullbrich, Y. L. Fan, M. Tropiano, K. A. McGarry, O. Zeika, M. K. Riede, C. J. Douglas, S. Barlow, S. R. Marder, D. Neher, D. Spoltore, K. Vandewal, *Nat. Energy* **2017**, 2, 17053.

[S6] D. Qian, Z. Zheng, H. Yao, W. Tress, T. R. Hopper, S. Chen, S. Li, J. Liu, S. Chen, J. Zhang, X. K. Liu, B. Gao, L. Ouyang, Y. Jin, G. Pozina, I. A. Buyanova, W. M. Chen, O. Inganas, V. Coropceanu, J. L. Bredas, H. Yan, J. Hou, F. Zhang, A. A. Bakulin, F. Gao, *Nat. Mater.* **2018**, 17, 703.
